# Preoperative risk grade predicts the long-term prognosis of intrahepatic cholangiocarcinoma: a retrospective cohort analysis

**DOI:** 10.1186/s12893-020-00954-x

**Published:** 2021-03-06

**Authors:** Jianping Zhao, Yao Chen, Jingjing Wang, Jian Wang, Ying Wang, Songshan Chai, Yuxin Zhang, Xiaoping Chen, Wanguang Zhang

**Affiliations:** 1 Hepatic Surgery Center, Tongji Hospital, Tongji Medical College, Huazhong University of Science and Technology, No. 1095 Jiefang Avenue, Wuhan, 430030 Hubei China; 2 Department of Medical Ultrasound, Tongji Hospital, Tongji Medical College, Huazhong University of Science and Technology, Wuhan, China; 3 Department of Hepatopancreatobiliary Surgery Treatment Center, Taihe Hospital, Hubei University of Medicine, Shiyan, China; 4 Department of Pathology, Tongji Hospital, Tongji Medical College, Huazhong University of Science and Technology, Wuhan, China

**Keywords:** Intrahepatic cholangiocarcinoma, Systemic inflammatory response, Platelet-to-lymphocyte ratio, Albumin and prognosis

## Abstract

**Background:**

Cumulating evidence indicates that the systemic inflammatory response (SIR) plays a crucial role in the prognosis of various cancers. We aimed to generate a preoperative risk grade (PRG) by integrating SIR markers to preoperatively predict the long-term prognosis of intrahepatic cholangiocarcinoma (ICC).

**Methods:**

468 consecutive ICC patients who underwent hepatectomy between 2010 and 2017 were enrolled. The PRG and a nomogram were generated and their predictive accuracy was evaluated.

**Results:**

The PRG consisted of two non-tumor-specific SIR markers platelet-to-lymphocyte ratio (PLR) and albumin (ALB), which were both the independent predictors of overall survival (OS). Multivariate analysis showed that the PRG was significantly associated with OS (PRG = 1: hazard ratio (HR) = 3.800, *p* < 0.001; PRG = 2: HR = 7.585, *p* < 0.001). The C-index of the PRG for predicting survival was 0.685 (95% CI 0.655 to 0.716), which was statistically higher than that of the following systems: American Joint Committee on Cancer (AJCC) 8th edition (C-index 0.645), Liver Cancer Study Group of Japan (LCSGJ) (C-index 0.644) and Okabayashi (C-index 0.633) (*p* < 0.05). Besides, the C-index of the nomogram only consisting of the tumor-specific factors (serum carcinoembryonic antigen, carbohydrate antigen 19-9, tumor number) could be improved to 0.737 (95% CI 0.062–0.768) from 0.625 (95% CI 0.585–0.665) when the PRG was incorporated (*p* < 0.001).

**Conclusions:**

The PRG integrating two non-tumor-specific SIR markers PLR and ALB was a novel method to preoperative predicting the prognosis of ICC.

## Background

The intrahepatic cholangiocarcinoma (ICC) has been the second most common primary liver tumor after hepatocellular carcinoma, and accounts for 10% to 15% of all primary liver malignancies [1]. The incidence of ICC has been increased and may be as high as 1–2 per 100,000 persons [2, 3]. Complete surgical resection remains the only potentially curative treatment option [4]. Unfortunately, only about 20% to 40% of ICC patients have the chance to receive surgical resection and the prognosis is unsatisfactory, with a median survival ranging from 24 to 36 months [3, 5]. The long-term survival is worse for unresectable ICC patients, with 5-year survival rate less than 5% to 10% after diagnosis [6]. Although several studies have described some prognostic factors and developed the relative staging systems for classification of ICC to provide the information of anticipated long-term outcomes, no one has had the excellent prognostic discrimination of ICC [2]. What’s more, the majority of these factors are tumor-specific pathologic markers, which are only available after surgery [7, 8].

The systemic inflammatory response (SIR) could influence the occurrence, development and prognosis of cancer [9]. Cancer-related inflammation is currently recognized as the seventh hallmark of cancer [10]. Many studies have suggested that the prognostic SIR markers based on the circulating blood cells could predict the long-term outcomes of patients in various tumors [11, 12]. Among these SIR markers, the preoperative lymphocyte-to-monocyte ratio (LMR), neutrophil-to-lymphocyte ratio (NLR) and platelet-to-lymphocyte ratio (PLR) have been revealed to be associated with the prognosis of ICC [13, 14]. Besides, preoperative haemoglobin and serum albumin (ALB) levels are also reported as the prognostic predictors for long-term prognosis of cancers [11, 15]. However, there are few studies have evaluated the value of these markers as independent predictors analyzed together and the extent how to integrate them to strengthen the predictive ability for ICC patients.

Therefore, the objective of the present study was to generate a preoperative risk grade by integrating the prognostic SIR markers to preoperatively predict the long-term prognosis of ICC.

## Methods

### Study population

468 consecutive ICC patients who underwent curative liver resection at the Hepatic Surgery Center, Tongji Hospital, Tongji Medical College, Huazhong University of Science and Technology (HUST) from January 2010 to December 2017 were enrolled retrospectively in this study. The inclusion criteria were: (1) ICC diagnosed pathologically; (2) liver function of Child–Pugh class A or class B; (3) no distant metastasis; (4) no previous treatment of ICC; (5) no history of other malignancies; and (6) detailed and precise follow-up records. This study was approved by the Institutional Review Boards of Tongji hospital, Tongji Medical College, HUST.

### Surgical resection procedure

The indications for liver resection in our center included that the liver function was Child–Pugh class A or class B, the tumors was resectable based on the preoperative imaging and the residual liver volume was enough predicted by volumetric computed tomography (CT) [16]. All the operations were performed by experienced surgeons in open and laparoscopic hepatectomy. The intraoperative ultrasound was routinely performed to determine the tumor location and the relation to the major blood vessels. The Pringle maneuver was only used in cases with uncontrolled bleeding. Major resection was defined as equal to or more than three Couinaud segments resection.

### Data collection and follow-up

The clinicopathological data including the SIR parameters of all patients were collected at admission and retrospectively reviewed and analyzed. Generally, the patients would receive the surgical treatment for ICC within 1 week after admission. After discharge, all patients were undertaken regular follow-up examination of serum α-fetoprotein (AFP), carcinoembryonic antigen (CEA) and carbohydrate antigen 19-9 (CA19-9), liver function and ultrasonogrphy every 4–6 weeks, and chest radiography every 8–12 weeks during the first 2 years. Thereafter, the intervals changed to 3–6 months. Investigations with CT, magnetic resonance imaging (MRI) or positron emission tomography (PET) were performed if recurrence was suspected. Once the intrahepatic tumor recurrence was identified, repeated liver resection, salvage transplantation, local ablation, transarterial chemoembolization (TACE), ethanol injection or systemic chemotherapy was performed based on the status of recurrence and the liver function.

### Statistical analysis

All the data were analyzed with SPSS version 21.0 software (SPSS, Chicago, IL) and R version 3.5.1 (R Foundation for Statistical Computing, Vienna, Austria). Continuous variables were expressed as the median with interquartile range (IQR), and categorical variables were expressed as the number and percentage. Prior to analyzing, the levels of tumor markers, including AFP, CEA and CA19-9, were log_10_ transformed. Comparisons of continuous variables were performed using Mann–Whitney U-test or Welch’s ANOVA, while Pearson chi-square analysis or Fisher’s exact test were used to compare categorical variables. The overall survival rates (OS) were estimated by the Kaplan–Meier method and were compared with the log-rank test. After the univariate analysis, the significant variables associated with the OS were then used for multivariate analysis using the Cox’s proportional hazards model. In both the univariate analysis and multivariate analysis, all the continuous clinicopathological characteristics were analyzed as continuous variables without dichotomization. After that, the software X-tile (Yale University, New Haven, CT, USA) was performed to determine the optimal cut-off value of PLR and ALB. The variables independently associated with OS were used to create a nomogram by R software using “rms” package. Calibration plots were generated to examine the performance characteristics of the predictive nomogram. The Concordance index (C-index) was measured and compared between the nomogram-predicted and observed Kaplan–Meier estimates of survival probability. The C-index ranges from 0.5 (no predictive power) to 1 (perfect prediction) [17]. *p* < 0.05 was considered to be significant difference.

## Results

### Clinicopathological characteristics

The clinicopathological characteristics of the patients were summarized in Table 1. Totally 468 patients were enrolled in the present study. The median age was 58 (IQR: 51–65). There were more male patients (282, 60.3%) than female patients (186, 39.7%). Median LMR, NLR and PLR were 3.3 (IQR: 2.1–4.8), 2.4 (IQR: 1.7–3.7) and 154.8 (IQR: 102.0–245.3), respectively. 107 (22.9%) patients had more than one tumor and 208 (44.4%) patients had tumors with diameter larger than 5 cm. Patients with vascular invasion (VI) and lymph node metastasis were 100 (21.4%) and 158 (33.8%), respectively. Most of patients had moderate differentiated tumor (346, 73.9%). More than half of patients received major hepatectomy (256, 54.7%). 134 (28.6%) patients had intraoperative blood transfusion and 90 (19.2%) patients received adjuvant chemotherapy.

**Table 1:** Patients and clinicopathological characteristics

Variables	No. of patients	%
Age (median, IQR)	58 (51–65)	
Gender (male)	282	60.3
Smoking (yes)	132	28.2
Drinking (yes)	66	14.1
HBsAg (positive)	202	43.2
ALT (U/L) (median, IQR)	29 (16–59)	
ALB (g/L) (median, IQR)	38.4 (34.9–41.0)	
TBIL (μmol/L) (median, IQR)	13.4 (8.9–22.4)	
Child–Pugh grade (B)	126	26.9
Cirrhosis (yes)	222	47.4
LMR (median, IQR)	3.3 (2.1–4.8)	
NLR (median, IQR)	2.4 (1.7–3.7)	
PLR (median, IQR)	154.8 (102.0–245.3)	
AFP (μg/L) (median, IQR)	3.3 (2.3–10.7)	
CEA (μg/L) (median, IQR)	2.8 (1.7–4.4)	
CA19-9 (U/mL) (median, IQR)	74.3 (21.9–590.4)	
Tumor number (multiple)	107	22.9
Tumor diameter (> 5 cm)	208	44.4
VI (yes)	100	21.4
Lymph node metastasis (yes)	158	33.8
Tumor differentiation		
Well	36	7.7
Moderate	346	73.9
Poor	86	18.4
Type of hepatectomy (major)	256	54.7
Blood transfusion (yes)	134	28.6
Adjuvant chemotherapy (yes)	90	19.2

*IQR* interquartile range, *HBsAg* hepatitis B surface antigen, *ALT* alanine aminotransferase, *ALB* albumin, *TBIL* total bilirubin, *LMR* lymphocyte to monocyte ratio, *NLR* neutrophil to lymphocyte ratio, *PLR* platelet to lymphocyte ratio, *AFP* α-fetoprotein, *CEA* carcinoembryonic antigen,* CA19-9* carbohydrate antigen 19-9, *VI* vascular invasion

### Development of the preoperative risk grade with SIR markers for preoperatively predicting the long-term prognosis of ICC

**Fig. 1:**
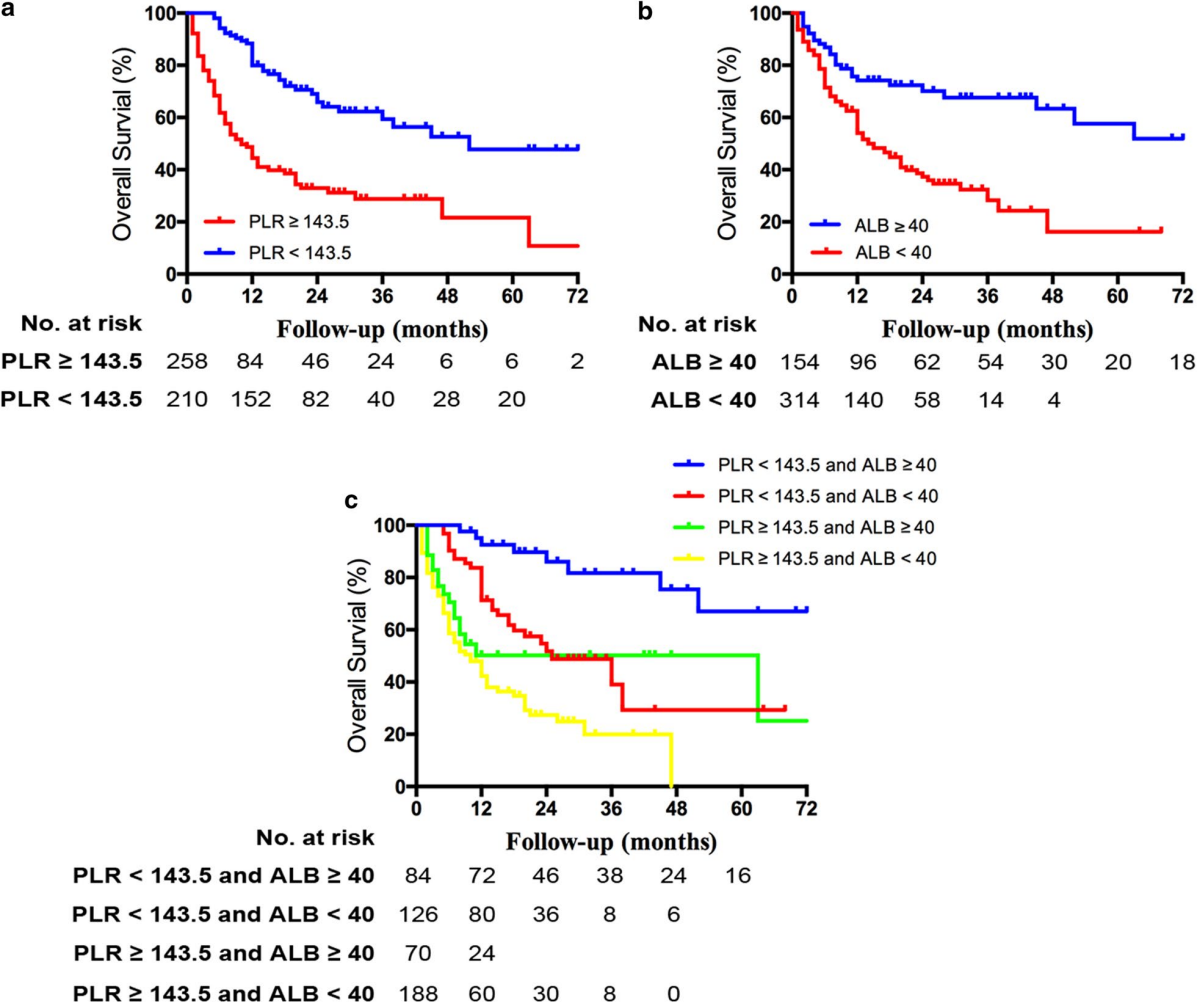
Kaplan–Meier analysis of patient survival with different levels of platelet to lymphocyte ratio (PLR), albumin (ALB) and the combination of them. **a** Overall survival (OS) between patients with PLR ≥ 143.5 and PLR < 143.5 (p < 0.001); **b** OS between patients with ALB < 40 g/L and ALB ≥ 40 g/L (p < 0.001); OS between the four subgroups with different combination of PLR and ALB (PLR < 143.5 and ALB < 40 g/L vs PLR ≥ 143.5 and ALB ≥ 40 g/L p = 0.066, all other p < 0.05)

The 1-, 3-, and 5-year OS of the patients were 60.7%, 43.1% and 31.3%, respectively. Univariate analysis showed that alanine aminotransferase (ALT), ALB, PLR, CEA and CA19-9, tumor number, VI and lymph node metastasis were significantly associated with the OS, while just the ALB, PLR, CA19-9, tumor number, VI and lymph node metastasis could independently predict the OS in multivariate analysis (Table 2). All those significant variables, except the ALB and the PLR, were tumor-related factors, and have been commonly considered to be associated with the long-term prognosis of ICC. The ALB and the PLR were two SIR markers, which could be detected preoperatively. Therefore, we tried to generate a preoperative risk grade (PRG) by integrating those two non-tumor-specific SIR markers to preoperatively predict the long-term prognosis of ICC. The optimal cutoff levels of the PLR and the ALB were 143.5 and 40.0 g/L, respectively, using the software X-tile. The OS was significant different between the different levels of the PLR, as well as the ALB (*p* < 0.001) (Fig. 1a, b). Thus, there were four subgroups when integrating the PLR and the ALB. Significant difference of OS existed among the four subgroups (*p* < 0.001), except between the subgroup PLR < 143.5 and ALB < 40 g/L and the subgroup PLR ≥ 143.5 and ALB ≥ 40 g/L (*p* = 0.066) (Fig. 1c). Therefore, we generated a PRG, which were defined as the follows: patients with PLR < 143.5 and ALB ≥ 40 g/L were defined as grade 0; patients with PLR < 143.5 and ALB < 40 g/L or PLR ≥ 143.5 and ALB ≥ 40 g/L were defined as grade 1; and patients with PLR ≥ 143.5 and ALB < 40 g/L were defined as grade 2 (see Additional file 1: Table S1). The patients with higher PRG had higher levels of NLR, PLR, AFP, CEA and CA19-9, and had more possibility of VI, and lymph node metastasis (all *p* < 0.05). Besides, the ALT, ALB, TBIL, Child–Pugh grade, LMR and tumor diameter were also associated with the PRG (all *p* < 0.001) (Table 3).

**Table 2:** Factors associated with the OS of ICC

Variables	Univariate analysis			Multivariate analysis		
	HR	95% CI	*p*	HR	95% CI	*p*
Age	0.988	0.977–1.00	0.051			
Gender (male v female)	0.909	0.701–1.118	0.474			
Smoking (yes v no)	0.886	0.664–1.181	0.409			
Drinking (yes v no)	1.161	0.807–1.671	0.421			
HBsAg (positive v negative)	0.942	0.726–1.222	0.654			
ALT (U/L)	1.001	1.000–1.003	0.037	1.001	0.997–1.002	0.440
ALB (g/L)	0.921	0.898–0.945	< 0.001	0.947	0.920–0.975	< 0.001
TBIL (μmol/L)	1.001	0.999–1.003	0.183			
Child–Pugh grade (B v A)	1.031	0.771–1.379	0.835			
Cirrhosis (yes v no)	1.202	0.930–1.553	0.161			
LMR	0.996	0.989–1.003	0.302			
NLR	1.005	0.975–1.037	0.733			
PLR	1.011	1.009–1.012	< 0.001	1.008	1.006–1.010	< 0.001
Log_10_ AFP	1.322	0.905–1.911	0.135			
Log_10_ CEA	1.678	1.345–2.093	< 0.001	1.013	0.755–1.359	0.933
Log_10_ CA19-9	1.615	1.428–1.827	< 0.001	1.475	1.278–1.703	< 0.001
Tumor number (multiple v single)	1.969	1.457–2.660	< 0.001	2.234	1.623–3.074	< 0.001
Tumor diameter (> 5 v ≤ 5 cm)	0.972	0.750–1.259	0.830			
VI (yes v no)	2.053	1.534–2.747	< 0.001	1.361	0.997–1.856	0.052
Lymph node metastasis (yes v no)	2.227	1.714–2.894	< 0.001	1.344	1.013–1.784	0.040
Tumor differentiation						
Well	Reference					
Moderate	1.016	0.598–1.725	0.954			
Poor	0.938	0.590–1.491	0.786			
Adjuvant chemotherapy (yes v no)	0.876	0.635–1.210	0.422			
Type of hepatectomy (major v minor)	1.058	0.818–1.369	0.668			
Blood transfusion (yes v no)	1.101	0.828–1.464	0.507			

*OS* overall survival rate, *ICC* intrahepatic cholangiocarcinoma, *HBsAg* hepatitis B surface antigen, *ALT* alanine aminotransferase, *ALB* albumin, *TBIL* total bilirubin, *LMR* lymphocyte to monocyte ratio, *NLR* neutrophil to lymphocyte ratio, *PLR* platelet to lymphocyte ratio, *AFP* α-fetoprotein, *CEA* carcinoembryonic antigen, *CA19-9* carbohydrate antigen 19-9, *VI* vascular invasion

**Table 3:** The relationship between the preoperative risk grade and the clinicopathological characteristics

Variables	Preoperative risk grade			*p*
	0 n = 84	1 n = 196	2 n = 188	
Age	58 (50–65)	59 (52–64)	58 (50–66)	0.623
Gender (male)	50 (59.5%)	112 (57.1%)	120 (63.8%)	0.404
Smoking (yes *v* no)	32 (38.1%)	54 (27.6%)	46 (24.5)	0.067
Drinking (yes)	16 (19.0%)	30 (15.3%)	20 (10.6)	0.150
HBsAg (positive)	46 (54.8%)	92 (46.9%)	64 (34.0%)	0.002
ALT (U/L)	21 (15–37)	29 (16–65)	36 (17–88)	< 0.001
ALB (g/L)	38.5 (35.6.2–41.3)	34.1 (33.2–38.2)	30.7 (29.20–34.6)	< 0.001
TBIL (μmol/L)	11.6 (8.7–15.4)	13.9 (9.1–22.5)	13.4 (8.9–82.5)	< 0.001
Child–Pugh grade (B)	4 (4.8%)	52 (26.5%)	70 (37.2%)	< 0.001
Cirrhosis (yes)	42 (50.0%)	82 (41.8%)	98 (52.1%)	0.114
LMR	4.4 (3.1–5.4)	3.4 (1.8–4.4)	2.6 (1.3–3.5)	< 0.001
NLR	1.97 (1.38–2.51)	2.3 (1.6–3.3)	3.1 (2.2–5.2)	< 0.001
PLR	87.4 (75.7–115.8)	127.7 (94.0–197.8)	234.7 (185.2–274.5)	< 0.001
Log_10_ AFP	0.66 (0.44–1.84)	0.51 (0.32–0.72)	0.51 (0.35–1.09)	0.020
Log_10_ CEA	0.29 (0.10–0.45)	0.49 (0.25–0.61)	0.49 (0.33–0.70)	< 0.001
Log_10_ CA19-9	1.56 (1.05–2.33)	1.91 (1.34–2.77)	2.21 (1.45–2.97)	< 0.001
Tumor number (multiple)	20 (23.8%)	42 (21.4%)	45 (23.9%)	0.821
Tumor diameter (> 5 cm)	50 (59.5%)	90 (45.9%)	68 (36.2%)	0.001
VI (yes)	6 (7.1%)	40 (20.4%)	54 (28.7%)	< 0.001
Lymph node metastasis (yes)	16 (19.0%)	54 (27.6%)	88 (46.8%)	< 0.001
Tumor differentiation				0.989
Well	6 (7.1%)	16 (8.2%)	14 (7.4%)	
Moderate	62 (73.8%)	146 (74.5%)	138 (73.4%)	
Poor	16 (19.0%)	34 (17.3%)	36 (19.1%)	
Adjuvant chemotherapy (yes)	22 (26.2%)	38 (19.4%)	30 (16.0%)	0.141
Type of hepatectomy (major)	48 (57.1%)	106 (54.1%)	102 (54.3%)	0.884
Blood transfusion (yes)	16 (19.0%)	58 (29.6%)	60 (31.9%)	0.088

*HBsAg* hepatitis B surface antigen, *ALT* alanine aminotransferase, *ALB* albumin, *TBIL* total bilirubin, *LMR* lymphocyte to monocyte ratio, *NLR* neutrophil to lymphocyte ratio, *PLR* platelet to lymphocyte ratio, *AFP* α-fetoprotein, *CEA* carcinoembryonic antigen, *CA19-9* carbohydrate antigen 19-9, *VI* vascular invasion

### Comparison of the predictive accuracy for OS between the PRG and three conventional staging systems of ICC

**Fig. 2:**
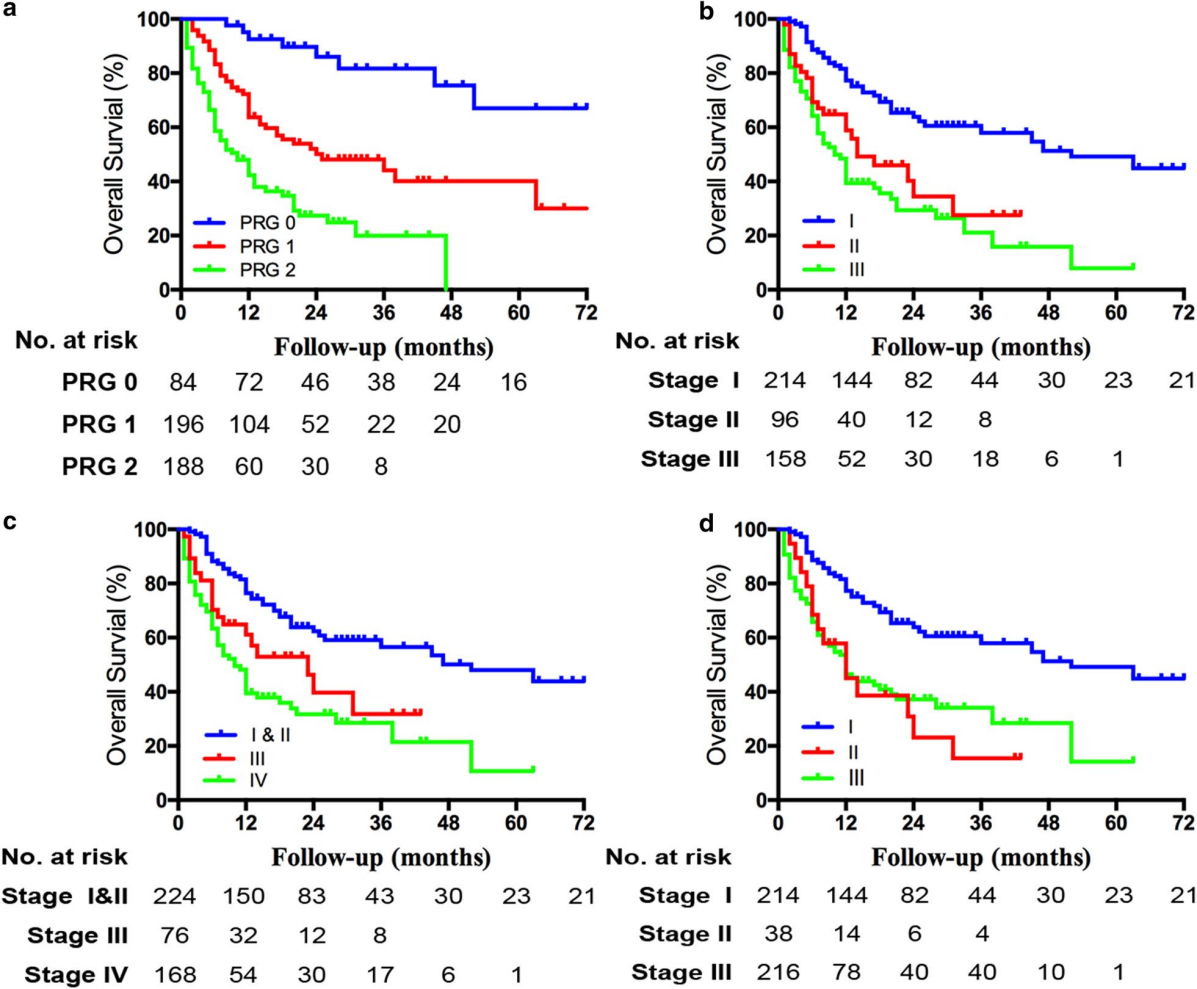
Kaplan–Meier analysis of patient survival according to different staging systems of ICC. **a** Preoperative risk grade (PRG), p < 0.001; **b** American Joint Committee on Cancer (AJCC) 8th edition, *p* < 0.05; **c** Liver Cancer Study Group of Japan (LCSGJ), *p* < 0.05; **d** Okabayashi, stage II v III *p* = 0.626

Kaplan–Meier analysis showed that a significant difference of OS exited among the three PRG subgroups (*p* < 0.001) (Fig. 2a). Besides, multivariate analysis revealed that the PRG was significantly associated with the OS (PRG = 1: hazard ratio (HR) = 3.800, *p* < 0.001; PRG = 2: HR = 7.585, p < 0.001) (Table 4). Both the American Joint Committee on Cancer (AJCC) 8th edition staging system (*p* < 0.05) (Fig. 2b) and the Liver Cancer Study Group of Japan (LCSGJ) staging system (*p* < 0.05) (Fig. 2c) showed good predictive accuracy for patients with different stages, while the OS showed no significant difference between the patients with stages II and stage III in the Okabayashi staging system (*p* = 0.626) (Fig. 2d). Furthermore, the C-index of the PRG for predicting the OS was 0.685 (95% CI 0.655 to 0.716), which was significantly higher than that of the AJCC 8th edition (C-index 0.645; 95% CI 0.612 to 0.679), the LCSGJ (C-index 0.644; 95% CI 0.611 to 0.678) and the Okabayashi (C-index 0.633; 95% CI 0.600 to 0.666) (*p* < 0.05). No significant difference of predictive accuracy was found among the three conventional staging systems (*p* > 0.05).

**Table 4:** The independent prognostic predictors for ICC

Variables	Univariate analysis			Multivariate analysis		
	HR	95% CI	*p*	HR	95% CI	*p*
ALT (≧ 40 v < 40 U)	1.366	1.053–1.772	0.019	1.221	0.932–1.600	0.148
CEA (≧ 5 v < 5 U)	1.857	1.372–2.513	< 0.001	1.449	1.049–2.000	0.024
CA19-9 (≧ 37 v < 37 U)	1.619	1.226–2.137	0.001	1.454	1.090–1.940	0.011
Tumor number (multiple v single)	1.969	1.457–2.660	< 0.001	2.529	1.849–3.460	< 0.001
VI (yes v no)	2.053	1.534–2.747	< 0.001	1.402	1.036–1.897	0.029
Lymph node metastasis (yes v no)	2.227	1.714–2.894	< 0.001	1.720	1.302–2.274	< 0.001
PRG						
0	Reference			Reference		
1	3.969	2.316–6.799	< 0.001	3.800	2.203–6.555	< 0.001
2	8.617	5.038–14.739	< 0.001	7.585	4.357–13.206	< 0.001

*ICC* intrahepatic cholangiocarcinoma, *ALT* alanine aminotransferase, *CEA* carcinoembryonic antigen, *CA19-9* carbohydrate antigen 19-9, *VI* vascular invasion, *PRG* preoperative risk grade

### Predictive nomogram for the long-term prognosis of ICC

**Fig. 3:**
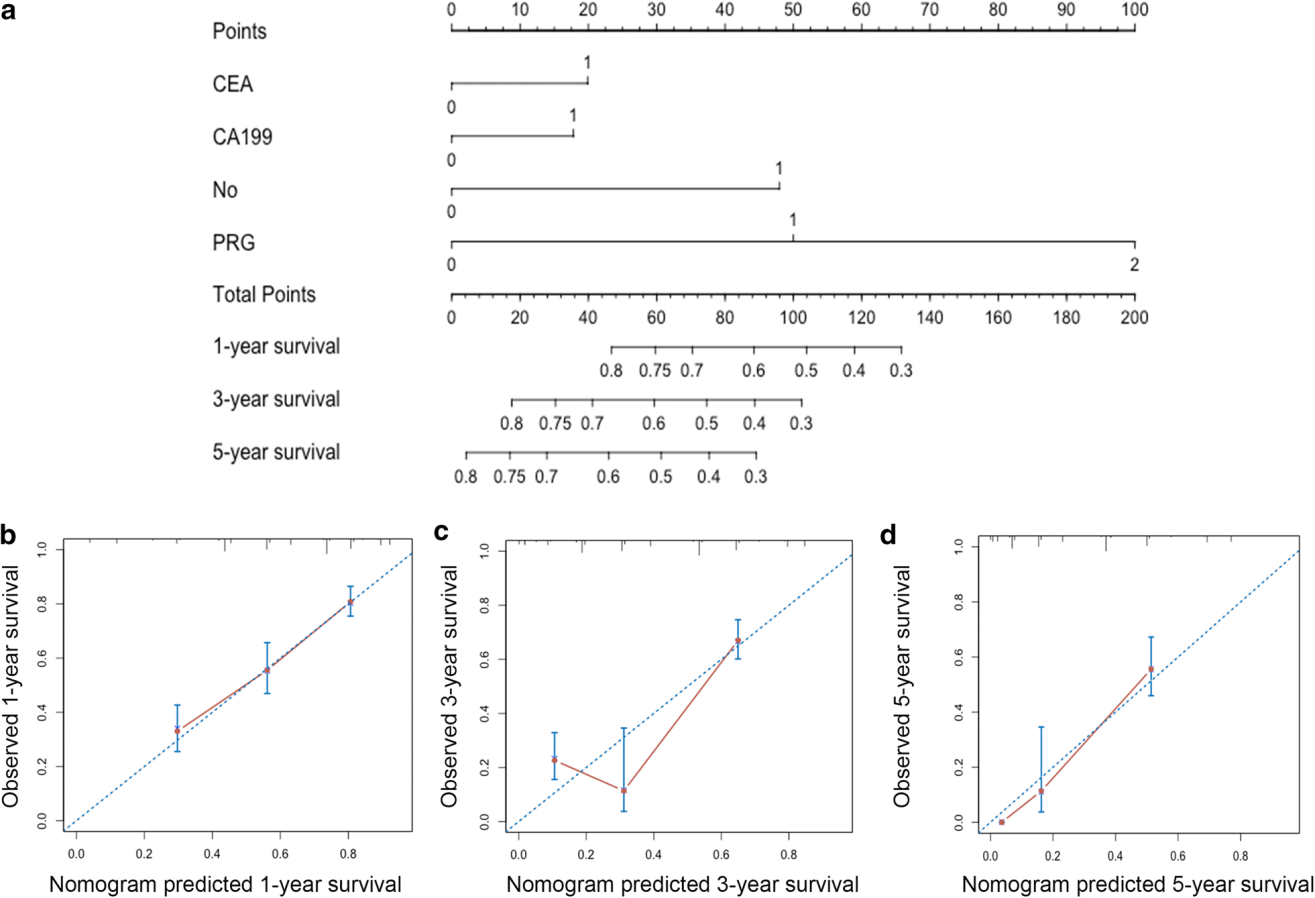
Nomogram for predicting 1-, 3- and 5-year overall survival (OS) of intrahepatic cholangiocarcinoma (ICC) patients. **a** Nomogram for predicting 1-, 3- and 5-year OS; calibration plot of nomogram for predicting patient survival at **b** 1-year, **c** 3-year and **d** 5-year. The 45-degree blue line represents the performance of the ideal model and the red line represents the performance of the proposed nomogram. Nomogram predicting OS is plotted on the x-axis and the actual OS is plotted on the y-axis. (*CEA* carcinoembryonic antigen, *CA199* carbohydrate antigen 19-9, *No* tumor number, *PRG* preoperative risk grade)

Cox’s proportional hazards model showed that the CEA, CA19-9, tumor number, VI, lymph node metastasis and the PRG were the independent prognostic predictors for ICC (*p* < 0.05) (Table 4). All those prognostic predictors, except the VI and lymph node metastasis, could be detected preoperatively, and were integrated to construct a nomogram for providing an effective way to preoperatively predict the long-term prognosis by a quantitative method (Fig. 3a). In internal validation, the calibration plots of the nomogram predicting 1-, 3- and 5-year survival performed well with the ideal model (Fig. 3b–d). Besides, the C-index of the multivariate prognostic model only consisting of the tumor-specific factors (CEA, CA19-9 and tumor number) could be improved to 0.737 (95% CI 0.062–0.768) from 0.625 (95% CI 0.585–0.665) when the PRG was incorporated (*p* < 0.001).

## Discussion

Surgery as the only effective treatment method could improve the long-term survival of patients with hilar cholangiocarcinoma and well-differentiated grading and R0 resection are significantly associated with a better outcomes for those patients [18]. Similarly, surgical resection remains the only potentially curative treatment option for ICC patients. However, only about 20% to 40% of the ICC patients are suitable to get surgical resection when diagnosed [3]. Even for these patients received hepatectomy, the long-term prognosis is still unsatisfactory with 5-year tumor recurrence rate 53% to 79% and the corresponding survival rate 23.0% to 35.2% [3, 4, 19]. In addition, major hepatic resection (54.7% in the present study) is commonly needed in resection of ICC and therefore associated with high risk of postoperative morbidity [8, 20]. Although several staging systems have been developed to predict the prognosis of ICC after liver resection, there still have some controversies over the development and the implementation of these models [4, 19]. Thus, accurately preoperative assessment of the long-term survival benefit from hepatectomy would be particularly important for preoperative patients selection.

As Virchow firstly described the links between cancer and inflammation in 1876, cumulating evidence has suggested that inflammation played an important role in tumors [9]. The SIR markers, such as C-reactive protein (CRP), ALB, LMR, NLR and PLR, have been reported as the independent prognostic predictors of various solid tumors [12, 21–25]. In the present study, we evaluated the relationship of clinicopathological characteristics and the long-term prognosis of 468 ICC patients, and found that the PLR and the ALB, two non-tumor-specific SIR markers, were significantly associated with OS. Further, we generated a novel PRG by integrating the PLR and the ALB after dichotomization, and found a significant difference of OS exited among the three PRG subgroups. Cox’s proportional hazards model showed that the PRG was an independent predictor of OS. Besides, the patients with higher PRG tended to have higher levels of CEA and CA19-9, and have greater possibility of VI and lymph node metastasis, all of which had been widely considered to be associated with poor prognosis of ICC and were the indications of and systemic therapy for ICC [3, 4, 26, 27]. Therefore, we believe that the ICC patients with high PRG should be advised to receive the neoadjuvant or prolonged systemic therapy to improve the long-term prognosis, although which need more studies to validate. Although several previous studies have reported the relationship between the PLR and hepato-pancreatico-biliary malignancies, only Chen and colleagues have reported that the PLR was an independent adverse prognostic factor for survival of ICC [13, 24, 28]. The ALB has been widely reported to be associated with the long-term outcomes of various cancers, including ICC [29, 30]. Inflammation-based scores consisting of CRP and ALB as the Glasgow prognostic score have been proven to be significantly associated with survival in various cancers [31, 32]. Besides, Saito and colleagues have reported that a prognostic scoring system, consisting of PLR, CRP, ALB and CEA, could predict the postoperative survival after resection of perihilar cholangiocarcinoma [29]. However, to our knowledge, the present study is the first time to evaluate the prognostic value of the PLR and the ALB in ICC.

To date, the conventional staging systems of ICC include the AJCC 8th, LCSGJ and Okabayashi. In the present study, all the three staging systems performed well in predicting the OS of ICC, except the Okabayashi staging system for no significant difference of OS existing between the patients with stages II and stage III. However, the prognostic prediction of ICC is traditionally based on the tumor-specific factors such as tumor number, tumor size, VI, lymph node metastasis and extrahepatic metastasis, some of which are only available after surgery. Whereas, the circulating platelet and lymphocyte of PLR, and serum albumin adopted in the PRG are routinely detected before surgery in clinical setting. Thus, PRG is an accessible and accurate method to preoperatively predict the long-term prognosis of ICC patients. A nomogram consisting of CEA, CA19-9, tumor number and PRG, which were the independent predictors of OS and could be detected preoperatively, were generated and performed well in internal validation for predicting the prognosis. The PRG played an important role in the nomogram with C-index improved from 0.68 to 0.75 when the PRG was incorporated.

The biological reason behind the prognostic value of PRG should be elucidate by the function of platelet, lymphocytes and ALB, respectively. The platelet, reported in previous studies, could promote the tumor-induced angiogenesis and invasiveness of tumor cells [33]. Besides, elevated blood platelet count might also reflect the tumor-induced SIR because the inflammatory mediators released in difference type of cancers could stimulate the proliferation of platelet progenitor cells [34, 35]. On the other hand, lymphocytes could enhance tumor immune-surveillance to inhibit tumor cell proliferation, invasion, as well as metastasis [36]. Several studies have suggested that the absolute lymphocyte count can predict the OS of various cancers [37]. Accordingly, an elevated circulating platelet count may reflect the progression of tumor and a low circulating lymphocytes count might be responsible for the impaired and insufficient host immune response to malignancy. Thus, high PLR is associated with poor prognosis in various cancers, including the ICC in the present study [24, 38]. It has been reported that the ALB level might correlate with the systemic inflammation [30]. Besides, the ALB is commonly used as the marker for assessing patient’s nutritional status [30]. Malignancy frequently causes patient malnutrition reflected in hypoalbuminemia, which may in turn affect the host immune response to tumors [30, 39]. Thus, hypoalbuminemia suggests the systemic inflammation as well as immune suppression, and therefore associated with the prognosis of various cancers [25, 30, 40, 41].

The SIR markers reflected the biological characteristics of tumors and could be used to predict the long-term prognosis whether the tumor is resectable or unresectable [42, 43]. Studies have showed that the systemic therapy including chemotherapy, radiotherapy and the chemoradiotherapy benefits the unresectable ICC [44–47]. More recently, the molecularly targeted therapy and immnuotherapy for ICC have achieved inspiring outcomes [48–51]. Therefore, we think the ICC patients with high PRG should receive more aggressive systemic therapy, no matter if they would have an operation. In our center, locoregional therapies such as transcatheter arterial chemoembolization (TACE), radioembolization, or external-beam radiation therapy (EBRT) would be recommended to the unresectable advanced-stage ICC. Of cause, the gemcitabine plus cisplatin therapy as the current first-line cytotoxic chemotherapy for advanced-stage cholangiocarcinoma would be considered firstly. At the same time, the target therapy combination with the immunotherapy might be recommended to unresectable ICC, especially for those patients with resistance to chemotherapy or actionable mutations [52, 53].

There are limitations in this study. First of all, this is retrospective study containing a small sample of 468 ICC patients from a single-center. Second, although there is no significant difference of ALB between the patients with and without cirrhosis (37.6 ± 4.6 g/L vs 38.1 ± 4.9 g/L, p = 0.220) in the present study, the cirrhosis and the situation that some patients with cirrhosis may have received albumin treatment before admission for surgery might have influenced the level of ALB. Third, the external validation should have been conducted to further verify the prognostic predicting value of the nomogram and PRG, although the nomogram incorporating PRG performed well in internal validation. Finally, the other systemic inflammation response marker such as CRP was not analyzed because it is not detected routinely in our center.

## Conclusions

We generated a novel prognostic predicting model PRG by integrating two non-tumor-specific SIR markers PLR and ALB. The PRG is an accessible and accurate method to preoperatively predict the long-term prognosis of ICC.

## Supplementary information

**Additional file 1: Table S1.** The definition of the preoperative risk grade.

## Data Availability

Data are available upon request from the corresponding author.

## Abbreviations

ICC: Intrahepatic cholangiocarcinoma
SIR: Systemic inflammatory response
LMR: Lymphocyte-to-monocyte ratio
NLR: Neutrophil-to-lymphocyte ratio
PLR: Platelet-to-lymphocyte ratio
ALB: Albumin
CT: Computed tomography
AFP: α-Fetoprotein
CEA: Carcinoembryonic antigen
CA19-9: Carbohydrate antigen 19-9 (CA19-9)
MRI: Magnetic resonance imaging
PET: Positron emission tomography (PET)
TACE: Transarterial chemoembolization
IQR: Interquartile range
OS: Overall survival
C-index: Concordance index
VI: Vascular invasion
ALT: Alanine aminotransferase
PRG: Preoperative risk grade
AJCC: American Joint Committee on Cancer
LCSGJ: Liver Cancer Study Group of Japan
