# Supplementary material for: Preoperative risk grade predicts the long-term prognosis of intrahepatic cholangiocarcinoma: a retrospective cohort analysis
Source: BMC Surg. 2021 Mar 6;21:113. doi: 10.1186/s12893-020-00954-x (PMC7936481; doi:10.1186/s12893-020-00954-x)
Supplement: Supplementary file 1 — Additional file 1: Table S1. The definition of the preoperative risk grade. [file 12893_2020_954_MOESM1_ESM.docx]

Additional Table S1. The definition of the Preoperative Risk Grade

| Variables | Preoperative Risk Grade |
| --- | --- |
| PLR (< 143.5) and ALB (≥ 40 g/L) | 0 |
| PLR (< 143.5) and ALB (<40 g/L) | 1 |
| PLR (≥ 143.5) and ALB (≥ 40 g/L) | 1 |
| PLR (≥ 143.5) and ALB (< 40 g/L) | 2 |

PLR, platelet to lymphocyte ratio; ALB, albumin.
